# Perceived Psychological and Physical Health as Predictors of Mortality and Quality of Life in Patients with Lymphedema: A Prospective Study Spanning Almost Two Decades

**DOI:** 10.1007/s12529-024-10340-z

**Published:** 2024-12-10

**Authors:** Katharina Loibnegger-Traußnig, Andreas R. Schwerdtfeger, Franz Flaggl

**Affiliations:** 1 Department of Psychology, University of Graz, Universitätsplatz 3, 8010 Graz, Austria; 2 Lymphatic Clinic, General Hospital Wolfsberg, Wolfsberg, Austria

**Keywords:** Lymphedema, Mortality, Quality of life, Anxiety, Depression

## Abstract

**Background:**

How does living with a chronic disease of the lymphatic system affect quality of life and mortality? Lymphedema is a chronic disease mostly affecting women and research is sparse. To this date, longitudinal studies evaluating biopsychosocial predictors for mortality and quality of life in lymphedema are largely missing. This study aims to identify possible predictors and could open innovative ways for treatment options.

**Method:**

Two hundred ninety-two patients with lymphedema partaking in a rehabilitation program in a lymphedema clinic were longitudinally assessed. The sample consisted of 86.2% women, aged between 18 and 83 years (*M* = 53.42, *SD* = 12.54), with a mean BMI (kg/m^2^) of 31.64 (*SD* = 8.26). Beginning with baseline assessment in 2002–2006, follow-up was evaluated 15–19 years later (*N* = 91). We assessed a variety of potential biopsychosocial predictors of mortality and quality of life (i.e., depression, anxiety, cancer). At follow-up, 19.5% of participants were deceased.

**Results:**

Age, gender, and cancer significantly predicted mortality (*R*^2^ = .27) and quality of life (*R*^2^ = .29). Anxiety and depression significantly predicted both quality of life and mortality when entered simultaneously. However, further analyses indicated suppressor effects and when entered separately, effects solely for depression or anxiety did not reach significance level.

**Conclusion:**

Age, gender, and cancer were the main predictors of mortality and quality of life in patients with lymphedema. Psychological predictors of mortality and quality of life were mainly due to suppressor effects, thus calling for caution when analyzing the contribution of mental health indicators for clinical outcomes.

**Trial Registration:**

This study was preregistered with the German Clinical Trials Register (Identifier DRKS00024450) and Open Science Framework (https://doi.org/10.17605/OSF.IO/RHXQJ).

## Introduction

Lymphedema is a chronic inflammatory disease distinguished through impaired lymphatic drainage [1] and is viewed as a global epidemic with millions of people affected [2]. Around 1.8% of the German population is diagnosed with lymphedema, while women are more frequently affected than men [3]. A distinction can be made between primary lymphedema and secondary lymphedema, whereas primary lymphedema is genetic [4] and secondary lymphedema occurs due to mechanical damage (e.g., surgery, accidents) or oncological treatments [4]. For example, approximately 25–90% of women suffer from chronic lymphedema following breast cancer [5] or up to 69% after gynecological cancer [6, 7]. A newer concept includes different forms of lymphedema and chronic swelling within the term *chronic edema*, accounting for the various affected body parts and different origins (e.g., lymphedema, venous edema, obesity, vascular malformations) [8]. A current study from the UK identified 3.93/1000 patients with chronic edema, recognizing almost twice as many women than men [9]. Although constituting a serious health concern, lymphedema is under-researched and undertreated [8, 10].

Patients with lymphedema often report symptoms and impairments on all biopsychosocial levels. On a psychological level, patients repeatedly report body image disturbance [11, 12] and high psychological distress [5, 13, 14]. A literature review concluded that patients with lymphedema experience greater levels of depression, anxiety, and poorer psychological adjustment, than the general population [15]. Further, patients regularly report relationship problems [11], trivialization by health care professionals [11, 13], insensitivity by people in the public, social isolation, and problems at work [13]. On a physical level, patients generally express symptoms such as pain, swelling, dry skin, and loss of functionality [11, 14, 16]. Further, patients with lymphedema are often classified as overweight (36.5%) or obese (39.3%) [17] and being diagnosed with both lymphedema and obesity increases risk for infections and hospitalizations as compared to having lymphedema within a normal weight range [18]. Besides hospitalization and infection rates, there is a hypothesized impaired lymphatic function provoked by obesity, linking obesity and lymphedema [19]. Overall, lymphedema can be expected to severely impact quantity and quality of life on various levels [2, 12, 14].

Up to date, there are—to the authors’ knowledge—only longitudinal studies on predicting quality of life but no longitudinal study available assessing biopsychosocial predictors of mortality in patients with lymphedema. A number of biopsychosocial variables have been recognized as predictors for mortality in the general population as well as in specific patient populations. BMI, depression, smoking, low education [20], self-rated health [20, 21], and quality of life [21] have all been considered major predictors for mortality in healthy individuals. Further, relationship status is considered a predictor for mortality, even though the effect seems to differ based on gender. In particular, being a single woman was significantly associated with higher all-cause mortality [22] and married men were half-likely to die than unmarried men in a 10-year follow-up [23]. In addition, a large longitudinal study corroborated results of psychosocial stress on mortality, suggesting that chronic stress could affect cardiovascular mortality negatively in middle-aged men [24]. Quality of life was also identified as an important predictor for mortality in patients with liver cirrhosis [25] and patients with cancer [26]. Further numerous studies showed that depression is an important predictor for mortality in patients with heart failure [27], organ transplants [28], and breast cancer [29], respectively. In contrast, the effects of anxiety on mortality risk were inconclusive and small in patients with organ transplants and cancer [27, 28]. On the other hand, a survey including the general population showed that depression and anxiety significantly increased mortality risk—people with anxiety and depression died 7.9 years earlier, than people without anxiety and depression [30].

Besides mortality, quality of life is an equally important outcome. A study in the general population with overall good quality of life showed that the main domains predicting overall quality of life were physical and psychological quality of life as well as depressive symptoms[31]. In longitudinal studies assessing cancer patients, lower levels of anxiety and depression [32, 33], younger age, and being married [34] were associated with better quality of life. Nevertheless, a longitudinal approach over a timespan of almost two decades on a broader spectrum on biopsychosocial predictors for mortality and quality of life in patients with lymphedema is still missing. As lymphedema is a chronic disease and accompanies patients mostly a large timespan of their life, it is from highest importance to evaluate those long-term effects.

## Hypotheses and Aim of the Study

This prospective study aimed to assess biopsychosocial predictors of mortality and quality of life in patients with lymphedema. It was hypothesized that low quality of life, not being married, older age, being male, high levels of anxiety, depression, and a higher BMI at baseline could predict mortality and quality of life at follow-up.

## Methods

### Study Overview

This study aimed to predict mortality and quality of life in patients with lymphedema in a longitudinal design. By controlling for the stable, trait-like aspects of quality of life, we aimed to isolate the residualized change, allowing us to examine the factors that contribute to variations in quality of life over time. Thus, data collection was accomplished at two timepoints. Baseline assessment was done in 2002–2006 and follow-up almost two decades later. Recognizing that lymphedema is a lifelong chronic condition, we aimed to implement a more extended follow-up period. Despite the moderate sample size, the extended follow-up period enhances the statistical power to accurately predict both quality of life and mortality. Achieving robust and meaningful results requires a sufficiently high incidence of mortality and a significant decline in quality of life over this longer duration.

This study was preregistered on Open Science Framework (https://doi.org/10.17605/OSF.IO/RHXQJ) and in the German Clinical trial register (DRKS00024450). Further, this study was approved by the Ethics Committee of the University of Graz (GZ. 39/36/63 ex 2020/21) and the State of Carinthia (S2021-10).

### Participants

Participants were 310 patients with lymphedema partaking in a stationary rehabilitation program for lymphedema treatment. The Lymphatic Clinic provides a 3-week in-patient rehabilitation program for patients diagnosed with lymphedema and an Austrian health insurance. Patients are referred through general practitioners and receive an interdisciplinary in-patient hospital setting rehabilitation, which is further mentioned to as stationary rehabilitation. Inclusion criteria at baseline were being diagnosed with primary or secondary lymphedema, over 18 years old, and German-speaking. Diagnoses were confirmed through a physician specialized in internal medicine and lymphology, through clinical exams (e.g. Stemmer sign), extensive anamnesis, and circumferential and volume measurement (e. g. Kuhnke measurement). At baseline, the study sample consisted of 86.2% women and 13.8% men, matching previous studies [17, 35] and prevalence data [3, 8]. Participants were between 18 and 83 years old (*M* = 53.42, *SD* = 12.54), with a BMI (kg/m^2^) ranging between 19.4 and 75.8 (*M* = 31.64, *SD* = 8.26). At follow-up, 19.5% of participants were deceased. Further demographic data are depicted in Table 1.

**Table 1:** Sociodemographic characteristics of participants at baseline split into deceased and alive patients at follow-up

	Alive		Deceased	
	*n* = 235		*n* = 57	
	*M*	*SD*	*M*	*SD*
Depression	0.56	0.53	0.54	0.47
Anxiety	0.43	0.46	0.33	0.40
BMI	31.49	8.20	32.35	8.77
Age	51.78	11.33	61.43	12.13
	In percent %			
Gender				
Women	88.7%		75%	
Cancer				
Yes	46%		63%	
Marital status				
Yes	61.7%		55%	
Education				
Apprenticeship	67.7%		71.7%	
High school	22.6%		20%	
College/university	9.7%		8.3%	

BMI = kg/m^2^, SQL-90-R was used to assess depression and anxiety on a 5-point rating scale from 0 = never to 4 = very strong

### Sampling Procedures

**Fig. 1:**
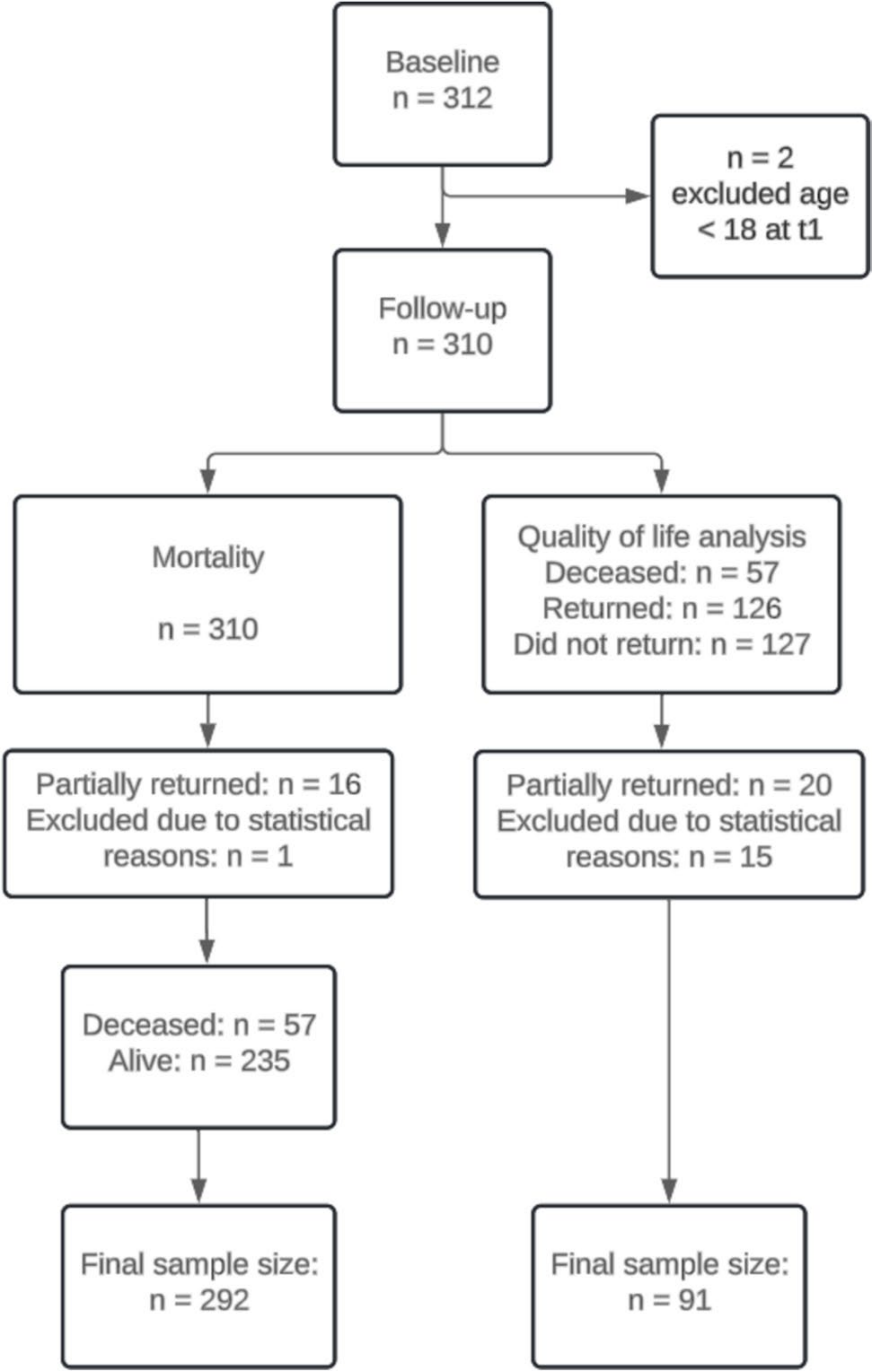
Flowchart of participants

Since the first data collection took place approximately 15–20 years ago and the average age of the participants ranged from 18 to 83 years (*M* = 53.42, *SD* = 12.54), it was reasonable to assume that there was a relatively high number of individuals who have since deceased. After assessing survival, participants were contacted via the Lymphatic Clinic at follow-up and informed about the procedure and the aim of the study. After informed consent was obtained, questionnaires were sent out and returned. All outcomes (depression, anxiety, quality of life, self-assessed health, and physical complaints) were assessed at baseline and at follow-up. A flowchart of the sample is depicted in Fig. 1. As sample size was determined due to the existing data set from pre-assessment, we were not able to determine sample size a priori; therefore, we calculated post hoc sensitivity analysis via G*Power [36, 37]. With 80% statistical power, we reached a minimal detected *odds ratio* for logistic regression of 0.61 or 1.39, which can be interpreted as small effect sizes. Power for linear regression was 82% for quality of life analysis to detect a medium effect [32–34], eventually reaching sufficient precision of the estimated effects. Further, the minimum detected effect size for linear regression was *f*^2^ = 0.17, depicting a small- to medium-sized effect.

### Outcomes

#### Mental Health

To assess depression and anxiety, the symptom checklist (SCL-90-R) was used. The SCL-90-R represents a standardized, valid, and reliable test procedure for measuring psychosomatic complaints [38] consisting of 90 items and nine scales. For this study, we used two out of the nine scales—depression and anxiety. Participants are asked to rate symptoms over the last 7 days (e.g., “tendency to cry,” “the feeling of not being interested in anything”) on a 5-point rating scale from 0 = never to 4 = very strong. For depression, internal consistency (McDonald’s *ω*) ranged between 0.87 (baseline) and 0.88 (follow-up), thus suggesting good reliability. For anxiety, McDonald’s *ω* was 0.81 for baseline and 0.85 for follow-up assessment. Overall, reliability of assessment of mental health indicators appeared good.

#### Self-Assessed Health

To evaluate self-assessed health, a one-item measure was used [39]. Participants were asked to rate their overall health on a 5-point rating scale ranging from 0 = bad to 4 = excellent. Mean score for the whole sample was 2.61 (*SD* = 1.09).

#### Physical Complaints

The Gießener Beschwerdebogen (GBB-24;[40]) was used to assess physical complaints. The GBB-24 is a standardized, valid, and reliable instrument for assessing subjective physical complaints [41] consisting of five scales (e.g., exhaustion, gastrointestinal complaints, cardiovascular complaints and musculoskeletal complaints, overall pain). Moreover, the total score *overall pain* aggregates all physical symptoms, which was considered the main measure in this research. Participants are asked to indicate the prevalence of symptoms (e.g., “back pain,” “nausea,” “fatigue,” “dizziness”) on a 5-point rating scale from 0 = never to 4 = strong. Internal consistency (McDonald’s *ω*) was 0.85 for baseline and 0.84 for follow-up, thus suggesting good reliability.

#### Quality of Life

The quality of life scale was used from Basic Documentation in Psychotherapy (Psy-BaDo)[42]. It is a standardized, valid, and reliable questionnaire for assessing quality of life in eight domains (health, work, finances, leisure time, social life, family, personally, sexual satisfaction). Participants are asked on a 7-point rating scale from 0 = very satisfied to 6 = not satisfied, to rate their quality of life in the mentioned domains (e.g., “I am satisfied with my physical health,” “I am satisfied with my social life”). A sum score was calculated across domains. Internal consistency of McDonald’s *ω* ranged from 0.72 for baseline to 0.68 for follow-up, thus suggesting fair reliability.

#### Mortality

In Austria, mortality is recorded by means of queries in the Central Civil Status Register (ZPR). This allows reliable information to be provided on the death of individuals. Hence, it was possible to gather reliable data on all participants taking part in this study. Information on cause of death and exact date of death was not available. Query for mortality was conducted in August of 2021.

### Statistical Methods

Statistical analyses were conducted using the software SPSS 28 for iOS [43]. Descriptive statistics were computed for all variables. Logistic regression was used to predict mortality and linear regression to analyze associations with quality of life. Predictors were gender, age, previous cancer disease, marital status, BMI, education, self-rated health, depression, anxiety, and quality of life at baseline. Odds ratio was used to quantify effect sizes for mortality analysis and beta weights as effect sizes for linear regression for quality of life. The level of significance was fixed at *p* < 0.05 (two-sided).

For model fit of logistic regression and linear regression analyses, all DF Beta, to check for cases with a large influence on the regression parameter, were < 1 [44]; thus, all cases were within those boundaries. Multicollinearity was checked in a first step through correlations among predictor variables, and in a second step VIF was calculated. VIF was < 10 [44] in all predictors, thus suggesting no severe multicollinearity in both models. Scatterplots were analyzed to check for heteroscedasticity. When checking for outliers, multiple methods were used. No outliers were found in both models, by making sure that less than 5% of cases standardized residuals were > 2 and no standardized residuals were > 3; further, Cook’s distance, to estimate influential points, was < 1 [44]. Mahalanobis distance was used to estimate outlying cases from the means of the predictor variables. Cut off (18.31) was calculated with chi squares at *p* < 0.05 and influential cases were excluded from analyses.

For model fit of logistic regression analyses, a model checking for linearity of logit was calculated and all interactions for continuous variables were *p* < 0.05. Thus, suggesting linear relationships between continuous variables and their logits.

## Results

### Descriptive Statistics

Descriptive data for both alive and deceased patients are reported in Table 1. At baseline, 38% of patients had clinically unremarkable scores in the depression scale, 31.4% had mild symptoms, and 30.6% of patients had values that were indicative for treatment, implying moderate to severe symptoms. 26.2% of patients had moderate to severe anxiety scores at baseline. At baseline, men (*M* = 0.30, *SD* = 0.27) had significantly lower depression scores than women (*M* = 0.52, *SD* = 0.44; *t*(100) = − 2.51, *p* = 0.02). Besides, no gender differences for anxiety at baseline and quality of life at both at baseline and follow-up were found (all *p* > 0.05). Quality of life at baseline (*M* = 46.00, *SD* = 5.76) was significantly higher than quality of life at follow-up (*M* = 40.67, *SD* = 11.80; *t*(90) = 3.63, *p* < 0.001).

At baseline, 83.5% of participants suffered from secondary lymphedema and 16.5% from primary lymphedema, all in stage 2. As depicted in Table 1, 46% of patients suffered from previous cancerous disease. Within those patients with a cancerous disease, 61.3% suffered from breast cancer, 31.4% from gynecological cancer, 4.4% from prostate cancer, 2.2% from sarcoma, and 0.7% from colon cancer.

As depicted in Fig. 1, a final sample size for mortality of *n* = 292 and quality of life of *n* = 91 was reached. At baseline, 312 participants were included in the sample size. In a first, step 2 participants were excluded due to age < 18 years. For mortality analysis, *n* = 16 were excluded due to only partially filling out the questionnaires and *n* = 1 was excluded for statistical reasons. In the final sample, *n* = 57 were deceased at follow-up and *n* = 235 were alive. For predicting quality of life at follow-up, 126 questionnaires were returned and 127 questionnaires were not returned, whereas the cause for non-returns remained unclear. We assumed that some participants might have changed their permanent residence or did not want to take part in the study. With that long follow-up, a return of more than one-third of the questionnaires seems appropriate.

### Correlations

As a first step, zero-order Pearson correlations between metric variables were calculated. Results are depicted in Table 2.

**Table 2:** Zero-order Pearson correlations for linear regression at baseline

	Age _t1_	BMI _(kg/m2) t1_	Depression _t1_	Anxiety _t1_	Pain _t1_	Quality of life _t2_	Quality of life _t1_	Self-assessed health _t1_
	*r*							
Age _t1_	1							
BMI _(kg/m2) t1_	0.16	1						
Depression _t1_	0.26*	0.13	1					
Anxiety _t1_	0.10	0.19	0.70**	1				
Pain _t1_	0.24*	0.34	0.53**	0.54**	1			
Quality of life _t2_	0.21*	0.09	0.12	− 0.21*	− 0.04	1		
Quality of life _t1_	0.13	− 0.26*	− 0.46**	− 0.28**	− 0.33**	− 0.17	1	
Self-assessed health _t1_	− 0.22*	− 0.36**	− 0.46**	− 0.39	− 0.49	− 0.02	− 0.37**	1

*N* = 91; *p < 0.05; **p < 0.001

### Predicting Mortality

To predict mortality at follow-up, gender, age, cancer, marital status, BMI, and education at baseline were entered into the model in step 1. At step 2, depression, anxiety, quality of life, overall pain, and self-rated health at baseline were added to the model. Detailed results are depicted in Table 3.

**Table 3:** Linear model of predictors of quality of life and logistic model of predictors of mortality with 95% confidence intervals

	Outcome: quality of life				Outcome: mortality					
Step 1	*B*	*SE*	*β*	*p*	*b*	*SE*	*p*	*OR*	*CI* lower	*CI* upper
Constant	34.13	10.55		0.002*	− 5.91	1.31	< 0.001*	0.00		
Gender _(male vs. female)_	0.42	0.17	0.28	0.016*	− 1.38	0.43	< 0.001*	0.25	0.11	0.59
Age _t1_	− 5.48	3.89	− 0.16	0.163	0.08	0.02	< 0.001*	1.08	1.05	1.12
Cancer _(no vs. yes)_	− 5.84	2.87	− 0.25	0.045*	0.93	0.36	0.010*	2.52	1.24	5.12
Married _(no vs. yes) t1_	− 0.97	2.70	− 0.04	0.720	− 0.36	0.33	0.285	0.70	0.36	1.35
BMI _(kg/m2) t1_	− 0.19	0.23	− 0.11	0.399	0.02	0.02	0.221	1.03	0.99	1.07
Apprenticeship vs. high school	1.63	3.21	0.06	0.613	0.32	0.43	0.452	1.38	0.60	3.19
Apprenticeship vs. college	− 8.32	4.50	− 0.21	0.068	− 0.59	0.62	0.341	0.55	0.16	1.86
Step 2	*B*	*SE*	*β*	*P*	*b*	*SE*	*p*	*OR*	*CI* lower	*CI* upper
Constant	50.10	16.44		0.003*	− 5.71	1.86	< 0.001*	0.003		
Gender _(male vs. female)_	0.39	0.18	0.26	0.033*	− 1.30	0.46	< 0.001*	0.27	0.11	0.68
Age _t1_	− 3.47	3.83	− 0.10	0.368	0.09	0.02	< 0.001*	1.09	1.05	1.13
Cancer _(no vs. yes)_	− 4.39	2.91	− 0.19	0.135	0.96	0.37	0.009*	2.62	1.27	5.38
Married _(no vs. yes) t1_	0.59	2.64	0.02	0.823	− 0.44	0.35	0.208	0.65	0.33	1.27
BMI (kg/m^2^) _t1_	− 0.09	0.24	− 0.05	0.711	0.03	0.02	0.245	1.03	0.98	1.07
Apprenticeship vs. high school	− 0.18	3.08	− 0.01	0.954	0.19	0.44	0.673	1.20	0.51	2.86
Apprenticeship vs. college	− 8.19	4.43	− 0.21	0.068	− 0.83	0.66	0.207	0.43	0.12	1.59
Self-rated health _t1_	0.42	1.97	0.03	0.830	0.04	0.15	0.787	1.04	0.78	1.38
Depression _t1_	10.80	4.91	0.36	0.031*	1.00	0.58	0.084	2.72	0.88	8.44
Anxiety _t1_	− 16.48	6.49	− 0.40	0.013*	− 1.47	0.70	0.037*	0.23	0.06	0.91
Quality of life _t1_	− 0.15	0.15	− 0.13	0.314	− 0.01	0.03	0.625	0.99	0.93	1.05
Pain _t1_	− 0.42	0.26	− 0.21	0.106	0.00	0.01	0.875	1.00	0.97	1.03

For linear regression of quality of life *N* = 91, for logistic model of mortality *N* = 292

Logistic regression analysis found significant predictors for mortality in patients with lymphedema, accounting for 17% of the variance (step 1: Cox & Snell *R*^2^ = 0.15 (Nagelkerke = 0.24), model *χ*^2^(7) = 47.77, *p* < 0.001; step 2: Cox & Snell *R*^2^ = 0.17 (Nagelkerke = 0.27), model *χ*^2^(12) = 54.19, *p* < 0.001). Specifically, gender, age, previous cancer disease, and anxiety were significant predictors for mortality. In the final model, we found that being female elevated the odds for mortality by 25%. Moreover, higher age predicted mortality by 9% and suffering from a previous cancerous disease at baseline more than doubled mortality odds by 2.62 times. In turn, being married, BMI, self-assessed health, depression, and education did not significantly contribute to mortality odds in patients with lymphedema. In contrast, elevated levels of anxiety attenuated mortality odds by 78%. Remarkably, there was a statistical trend, depicted in Table 3, (*p* = 0.084) of depression on mortality, although descriptive statistics between alive and deceased patients barely differed (Table 1). Together, with the high correlation between anxiety and depression at baseline (*r* = 0.70), those findings suggest suppressor effects of depression and anxiety on mortality.

### Predicting Quality of Life

To predict quality of life at follow-up, gender, age, cancer, marital status, BMI, and education at baseline were entered in step 1. At step 2, depression, anxiety, quality of life, overall pain, and self-rated health at baseline were added to the model to investigate predictors for quality of life.

Linear regression revealed significant predictors for quality of life, accounting for almost 30% of the variance (step 1: *R*^2^ = 0.14, *p* = 0.07; step 2: *R*^2^ = 0.29, *p* = 0.01). Precisely, gender significantly predicted quality of life at follow-up. Further, being male was associated with higher quality of life at follow-up. There was also a small effect of no previous cancer predicting better quality of life at follow-up. Adding psychosocial variables in step 2 further increased the amount of explained variance by 15% (*p* = 0.01). Specifically, anxiety had a negative effect on quality of life and, contrary to expectations, depressive symptoms were positively related to quality of life. Importantly though, considering results of zero-order correlations, depression was unrelated to quality of life at follow-up, while there was a high correlation with anxiety, thus again suggesting suppressor effects [45].

### Further Analyses Targeting Suppressor Effects

Due to previously assumed suppressor effects in both regression models, we conducted separate analyses for anxiety and depression to evaluate the trustworthiness of the findings.

#### Separate Models for Predicting Mortality

In a first step, depression was considered as a main predictor. The model explained 24% of the variance (step 1: Cox & Snell *R*^2^ = 0.15 (Nagelkerke = 0.24), model *χ*^2^(7) = 48.27, *p* < 0.001; step 2: Cox & Snell *R*^2^ = 0.16 (Nagelkerke = 0.25), model *χ*^2^(10) = 48.76, *p* < 0.001). Gender (*OR* = 0.27; *CI* 0.11, 0.66), age (*OR* = 1.09; *CI* 1.05, 1.13), and previous cancer disease (*OR* = 2.56; *CI* 1.26, 5.23) were significant predictors for mortality in patients with lymphedema, thus corroborating the results found in the original model. Of note, neither BMI, marital status, depression, self-assessed health, education, quality of life, nor overall pain (all *p* > 0.05) reached the level of significance.

Similar results were found for the final model including anxiety (step 1: Cox & Snell *R*^2^ = 0.15 (Nagelkerke = 0.24), model *χ*^2^(7) = 49.97, *p* < 0.001; step 2: Cox & Snell *R*^2^ = 0.16 (Nagelkerke = 0.26), model *χ*^2^(10) = 52.84, *p* < 0.001). Again, only gender (*OR* = 0.32, *CI* 0.13, 0.77), age (*OR* = 1.09, *CI* 1.06, 1.13), and previous cancer disease (*OR* = 2.40, *CI* 1.19, 4.82) were significant predictors for mortality. BMI, marital status, anxiety, self-assessed health, education, quality of life, and overall pain (all *p* > 0.05) did not reach significance level.

#### Separate Models for Predicting Quality of Life

The model using depression as a main predictor was also significant (step 1: *R*^2^ = 0.14, *p* = 0.07; step 2: *R*^2^ = 0.23, *p* = 0.03). Age (*β* = 0.34, *p* = 0.005), higher education (*β* = − 0.27, *p* = 0.020), and quality of life at baseline (*β* = − 0.23, *p* = 0.048) reached significance level for predicting quality of life at follow-up. Further, we found a statistical trend for previous cancer disease (*β* = − 0.25, *p* = 0.052) and overall pain at baseline (*β* = − 0.23, *p* = 0.073). In turn, gender, BMI, marital state, self-assessed health, and depression (all *p* > 0.05) did not reach level of significance.

The model using anxiety as a main predictor was significant (step 1: *R*^2^ = 0.14, *p* = 0.07; step 2: *R*^2^ = 0.24, *p* = 0.02). Age (*β* = 0.34, *p* = 0.004), higher education (*β* = − 0.23, *p* = 0.045), and quality of life at baseline (*β* = − 0.33, *p* = 0.007) reached significance level for predicting quality of life at follow-up. Neither previous cancer disease (*β* = − 0.19, *p* = 0.132) nor anxiety at baseline (*β* = − 0.19, *p* = 0.136) were significant. The same was true for gender, BMI, marital state, self-assessed health, and overall pain (all *p* > 0.05).

## Discussion

This study aimed to assess biopsychosocial predictors of both mortality and quality of life in patients with lymphedema over a time span of almost two decades. As could be expected, age, gender, and cancer were among the most robust predictors of both quality of life and mortality. Contrary to expected, relationship status, BMI, and self-rated health were not predictive of the outcomes. Moreover, when entered simultaneously, anxiety predicted better quality of life and lower risk for mortality, while depression was associated with worse outcomes, especially quality of life. However, anxiety and depression symptoms were strongly interrelated and when entered separately, neither anxiety nor depression predicted life quantity and quality. Hence, findings were due to suppressor effects, meaning that irrelevant variance in both concepts were suppressed, leading to inflated effect estimates.

Besides the trivial finding that older age predicted mortality, this study showed a significant effect of gender corroborating previous findings that men were at a higher mortality risk than women [46]. Further, suffering from cancer at baseline was associated with a significantly higher mortality rate at follow-up. After controlling for depression, quality of life at baseline was a significant predictor for quality of life at follow-up, thus suggesting that the suppressor effect diminished this effect.

### Suppressor Effects

The effects of mental well-being, operationalized through anxiety and depression, were varying throughout models, and suppressor effects were prominent. Thus, entering anxiety and depression as highly correlated together in one model, as is often the case (e.g., [47, 48]), must be considered faulty. For example, depression and anxiety correlated by *r* = 0.80 in lung cancer patients [49] and *r* = 0.78 in heart failure patients [47], thus fostering suppressor effects. Further, vague depiction of statistical analyses makes it difficult to recognize possible undetected suppressor effects in similar studies as zero-order correlations are not always reported or statistical details are missing [32, 48].

In conclusion, we advocate for caution when examining effects of both anxiety and depression as predictors for clinical outcomes. Like in the present study, suppressor effects are likely, given high intercorrelations between concepts. Based on our findings, we suggest conducting separate analysis for depression and anxiety when predicting mortality and quality of life in longitudinal studies.

### Body Weight

The absence of significant effects of BMI on mortality and quality of life in our study corroborates the obesity paradox [50–52]. Precisely, large studies showed that overweight does not increase the risk for mortality [53]. Further, higher BMIs were associated with a lower risk of death for heart failure patients [51, 52]. Consequently, we call for an evidence-based approach when dealing with patients with lymphedema and obesity and propose implementing a concept like “health-at-every-size” into weight management when treating obese patients with lymphedema. Health-at-every-size features various non-weight-centric strategies such as nutrition and physical activity–based approaches [54]. In a systematic review, health-at-every-size interventions showed promising effects on the cardiovascular status, eating behaviors, quality of life, and psychological well-being [55].

### Limitations and Implications for Further Research

Several limitations need to be addressed, when discussing this study. Firstly, we did not control for other important covariates, such as cardiovascular fitness and health-related behavior (e.g., alcohol consumption, smoking) at baseline, since these measures were not available then. Secondly, further rehabilitation stays after baseline were not controlled for, which might have influenced quality of life and mortality in the following years. Thirdly, due to data protection laws, it was not possible to gather information on precise time of death and cause of death.

Given that the psychosocial predictors included in this study did not successfully predict quality of life and mortality at follow-up, this points to the potential relevance of alternative predictors not accounted for. Particularly for predicting mortality, cardiovascular fitness [56], lifestyle behaviors (such as smoking) [57], or cognitive impairments [20] appear to have a significant impact. Moreover, resilience—an important determinant of quality of life [58, 59]—was not included as a baseline predictor, which may limit the depth of insights into long-term quality of life trajectories. When evaluating long-term outcomes in chronically ill patients, it is also important to consider response shift as a potential influence. Response shift describes the adjustment of internal standards, values, and perceptions of quality of life over time, a phenomenon frequently observed in chronically ill patients [60–63]. Consequently, response shift could impact the validity and reliability of long-term quality of life assessments in this population [63].

In addition to response shift theory, Golembiewski and colleagues [64, 65] propose three types of change: alpha, beta, and gamma change. Alpha change refers to the absolute change of a stable dimension that can be assessed through pre- and post-testing [64]. Beta change involves the subjective recalibration of these stable dimensions within the same framework of measurement from the respondents [64]. Gamma change entails a reconceptualization of psychological dimensions, reflecting a transformation in the underlying measured variable over time [64]. In psychological research, alpha changes are often anticipated; even though, beta and gamma changes tend to be more predominant [66]. As observed in this study, over the extended timespan between baseline and follow-up, beta and/or gamma changes are likely to be present, although only alpha changes were assessed.

Apart from that, this study’s main strength is the long duration of data collection as well as assessing various biopsychosocial predictor variables. Of note, the sample size of this study was sufficient for detecting small- to medium-sized effects as revealed by post hoc sensitivity analysis.

As to the best of the authors’ knowledge, this was the first study to longitudinally examine mortality and quality of life in patients with lymphedema. Hence, future research should aim to replicate this study’s findings with a larger data set and in respect of lymphedema type, cancer type, and lymphedema stage.

## Conclusion

This unique study was the first approach applying a time span of almost two decades with a reasonable sample size. Mainly, the findings suggest that common predictors for mortality, such as gender or previous cancer disease, must be considered robust predictors for quality of life and mortality in patients with lymphedema. With respect to psychological risk factors, we call for caution when interpreting mental health effects with a high proportion of shared variance potentially leading to suppressor effects. Moreover, the findings suggest that BMI seems to not severely impact quality of life and mortality in this specific sample, as often hypothesized [67, 68]. Since we are aware of a possible type II-error in the interpretation of this finding, we call for further studies to confirm this effect.

This study gave first insights into possible predictors for quality of life and mortality in patients with lymphedema in the long term. Although we could not find strong arguments toward a contribution of psychosocial predictor variables for both mortality and quality of life, further studies are certainly needed to disentangle psychosocial and behavioral effects in this specific sample to further improve treatment options.

## Data Availability

Data and code are available upon request.
